# Supplementary material for: A Nonlinear Simulation Framework Supports Adjusting for Age When Analyzing BrainAGE
Source: Front Aging Neurosci. 2018 Oct 24;10:317. doi: 10.3389/fnagi.2018.00317 (PMC6208001; doi:10.3389/fnagi.2018.00317)
Supplement: Supplementary file 1 [file Data_Sheet_1.docx]

Supplementary Material

A nonlinear simulation framework supports adjusting for age when analyzing BrainAGE

Trang T. Le†, Rayus Kuplicki†, Brett A. McKinney, Hung-wen Yeh, Wesley K. Thompson, Tulsa 1000 Investigators, Martin P. Paulus

*** Correspondence:** Rayus Kuplicki: rkuplicki@laureateinstitute.org

**Supplementary Figure S1.** Simple linear simulation, demonstrating the correlation between residuals and observed values. Here, $y=x+\varepsilon$ where $\varepsilon$ was normally distributed with mean 0 and standard deviation σ. After fitting a line, the residuals are correlated with observed values of y, and that correlation decreases with decreasing σ.

Low correlation Medium correlation High correlation


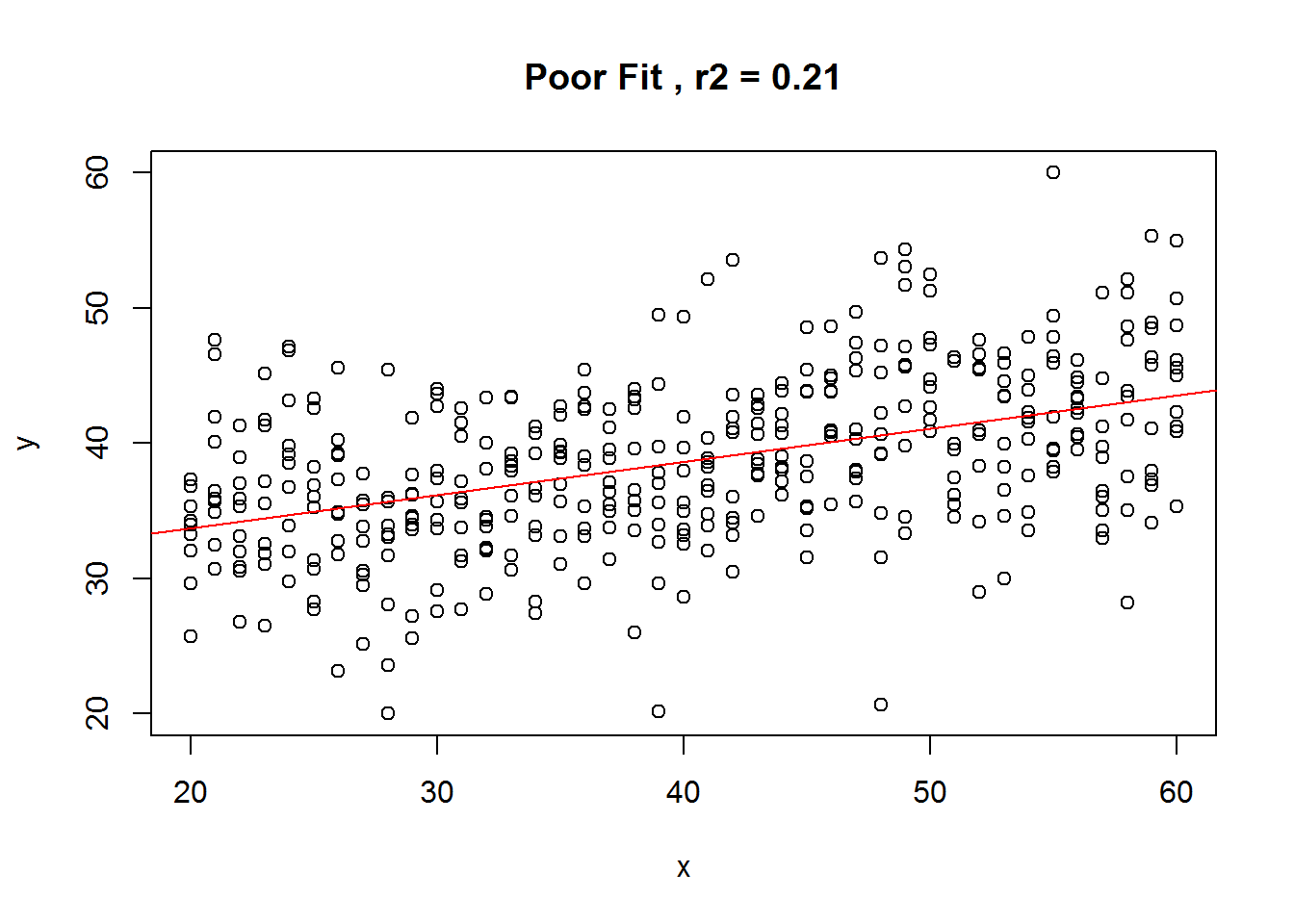

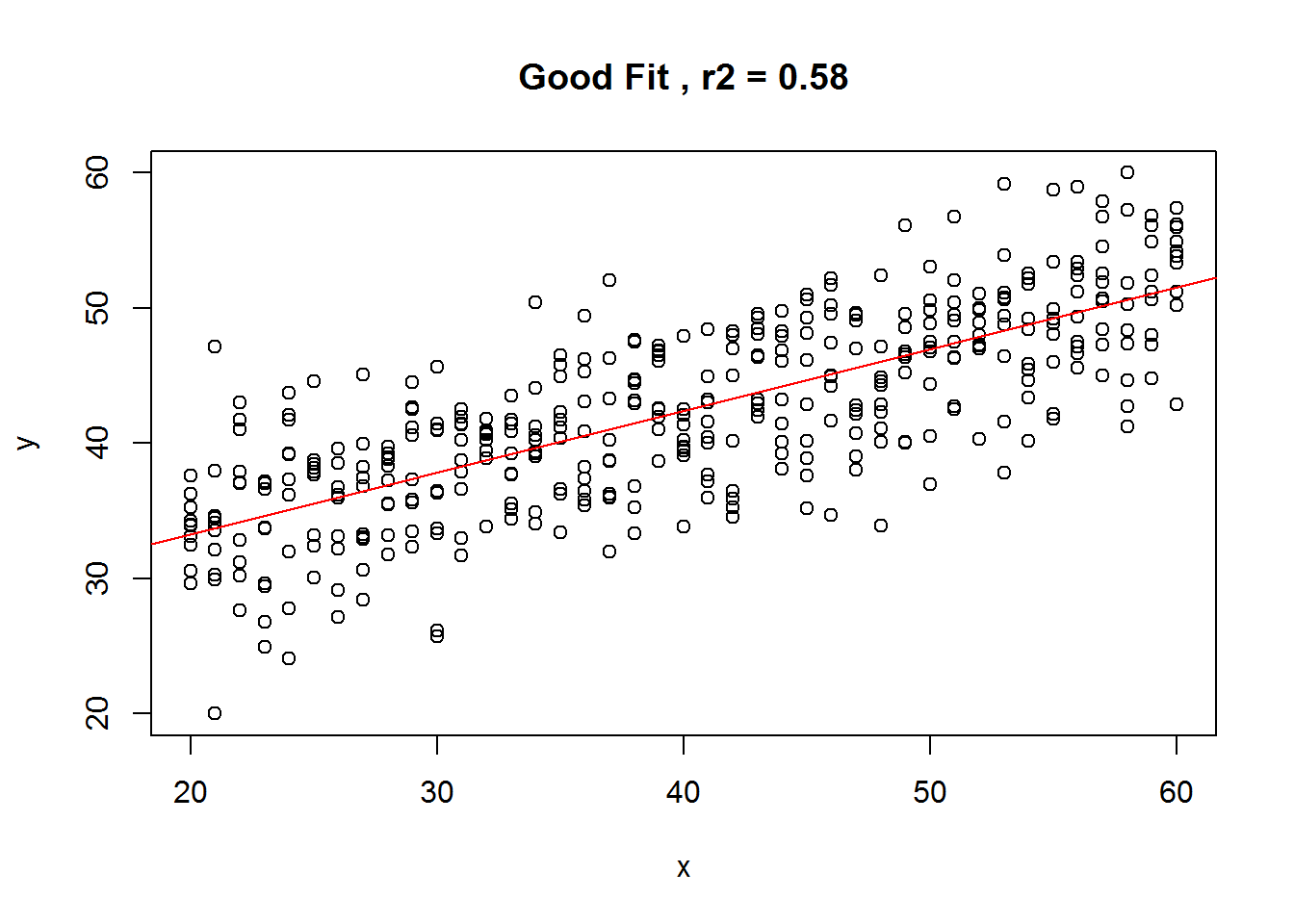

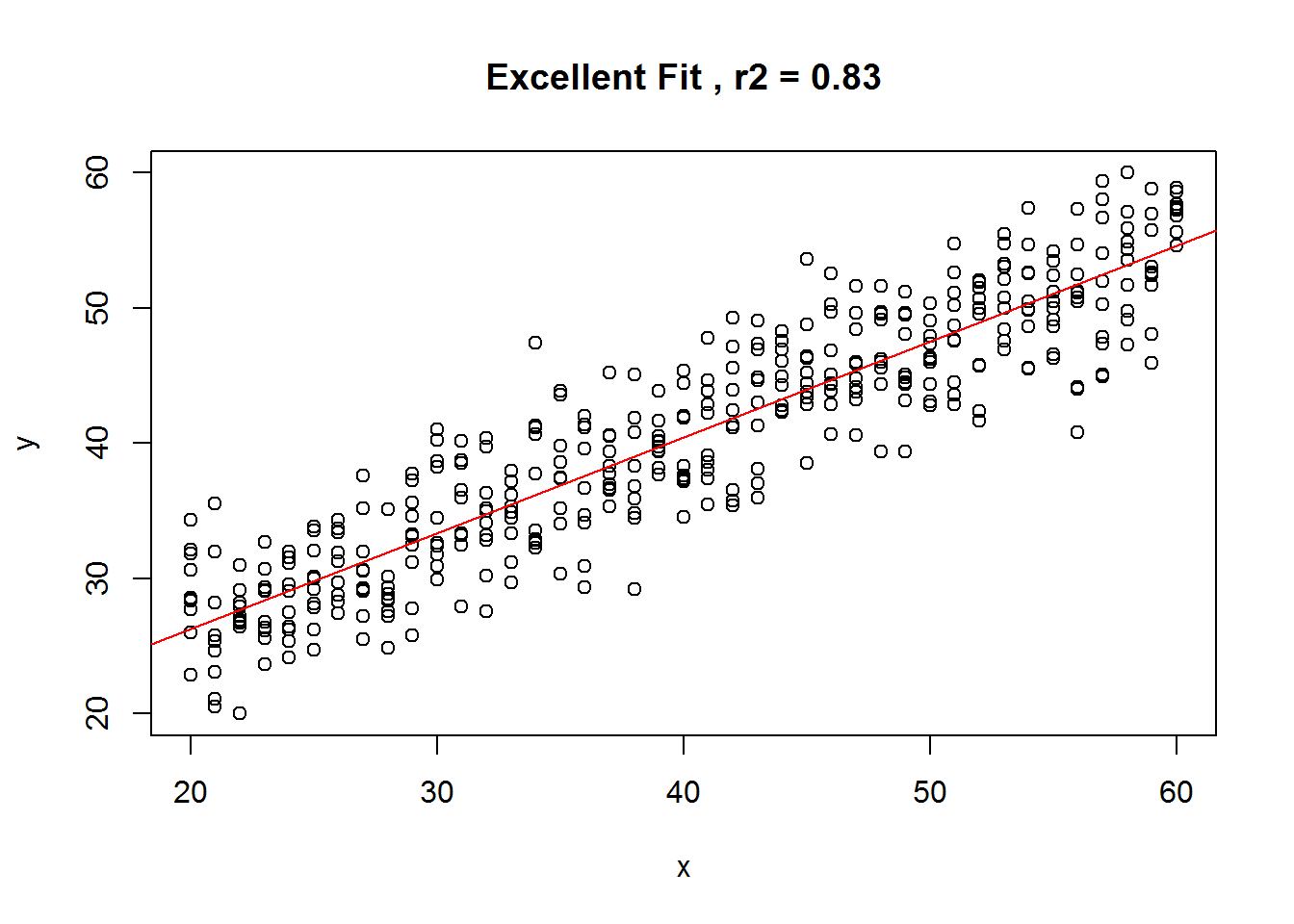


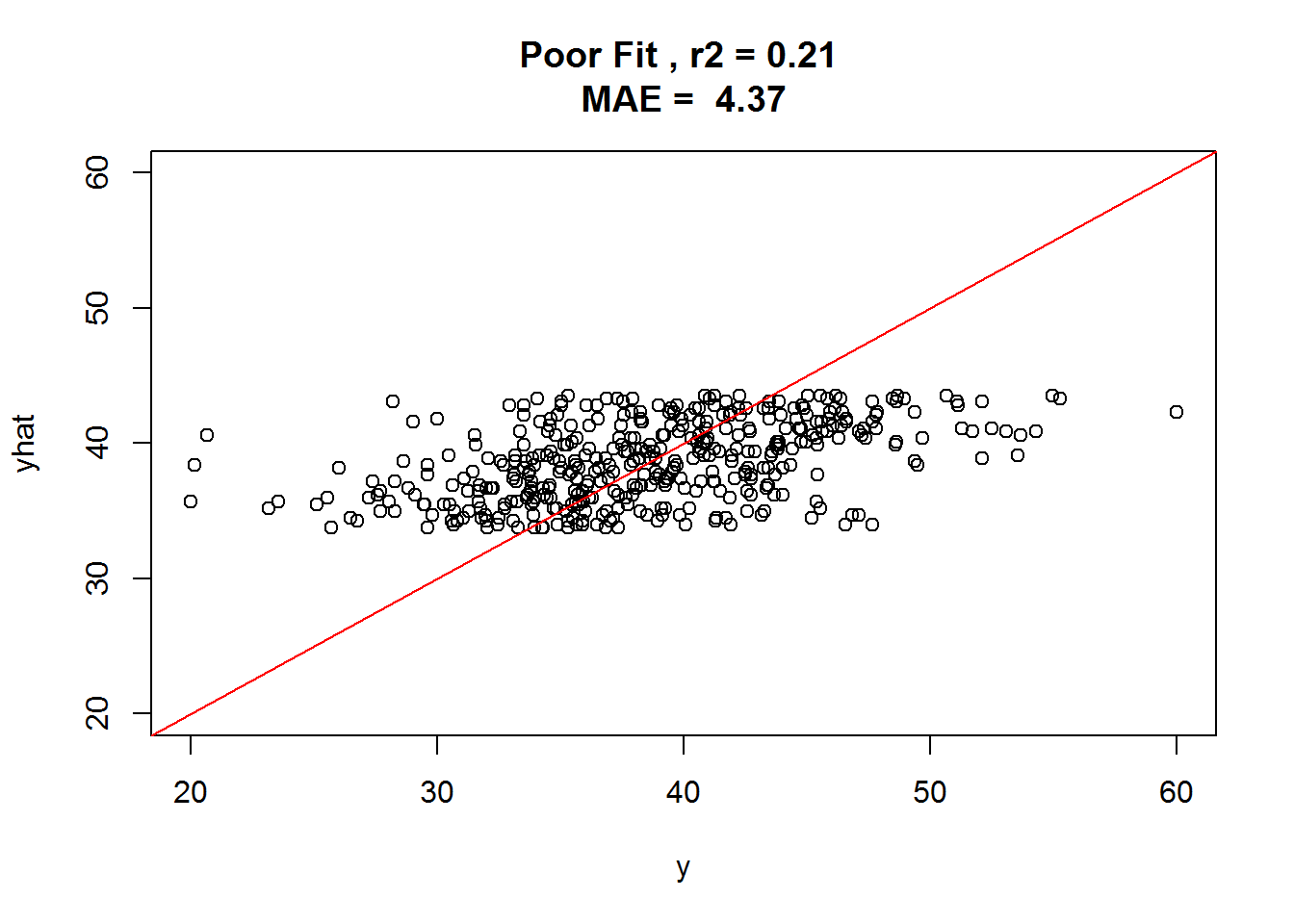

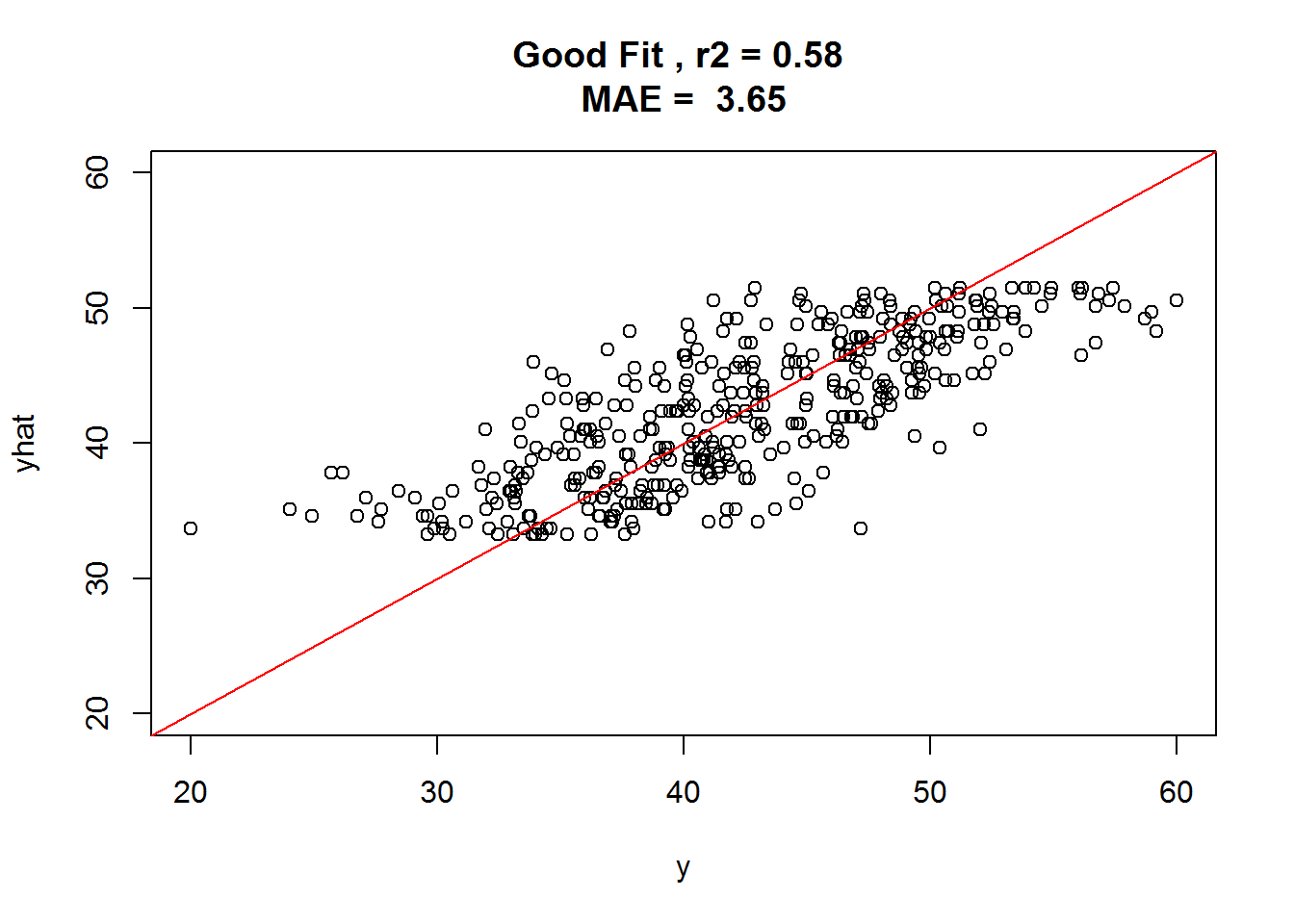

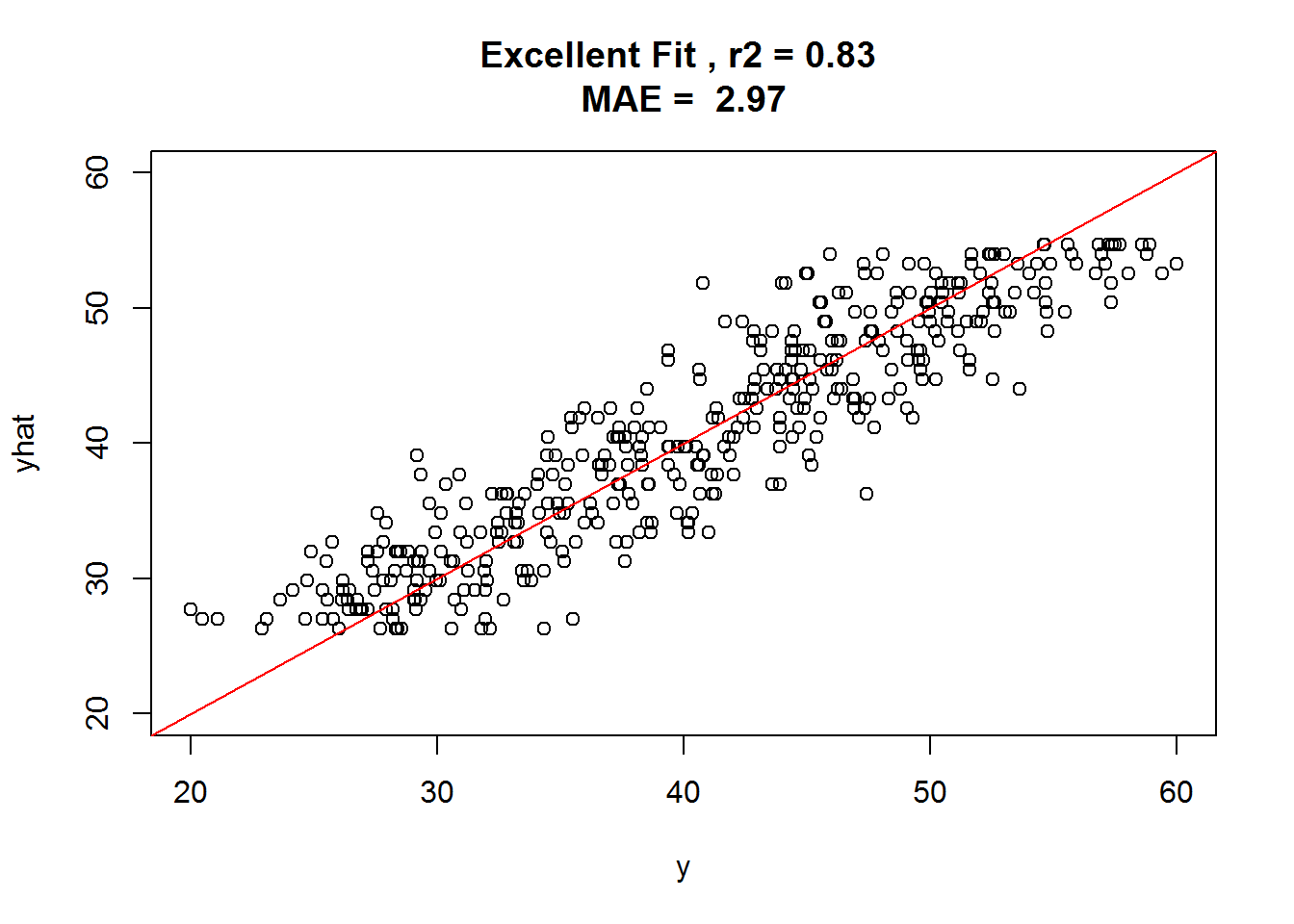


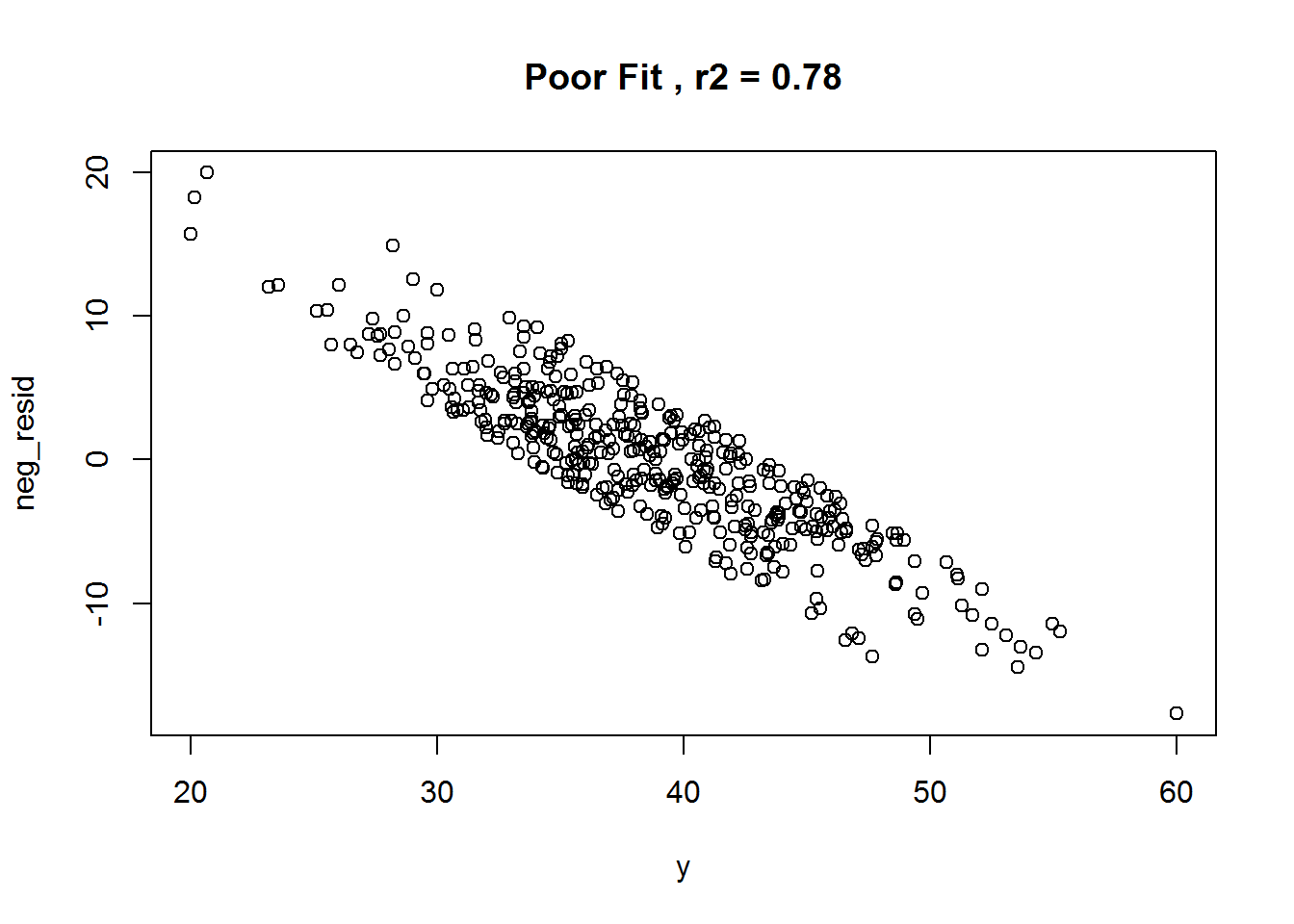

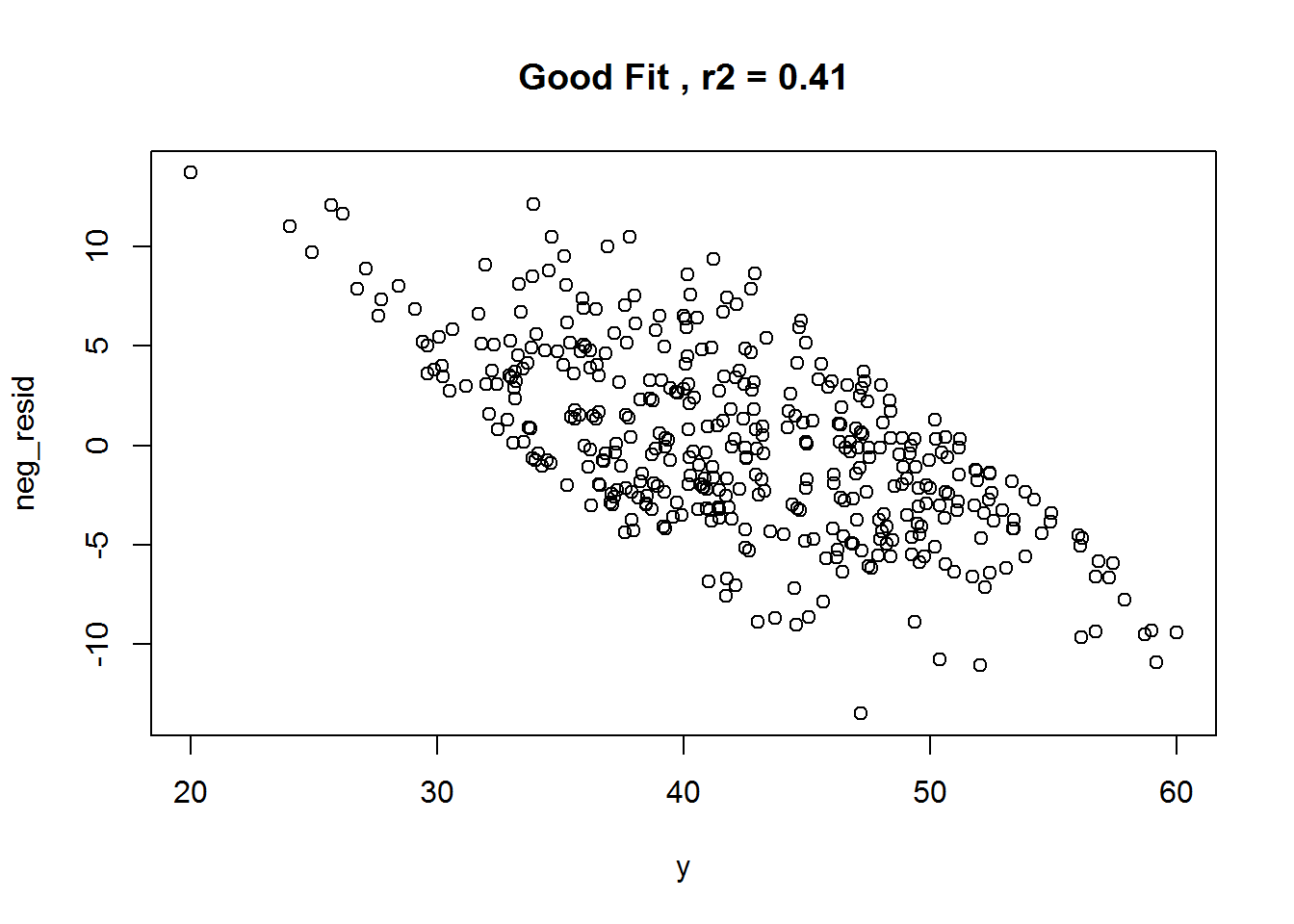

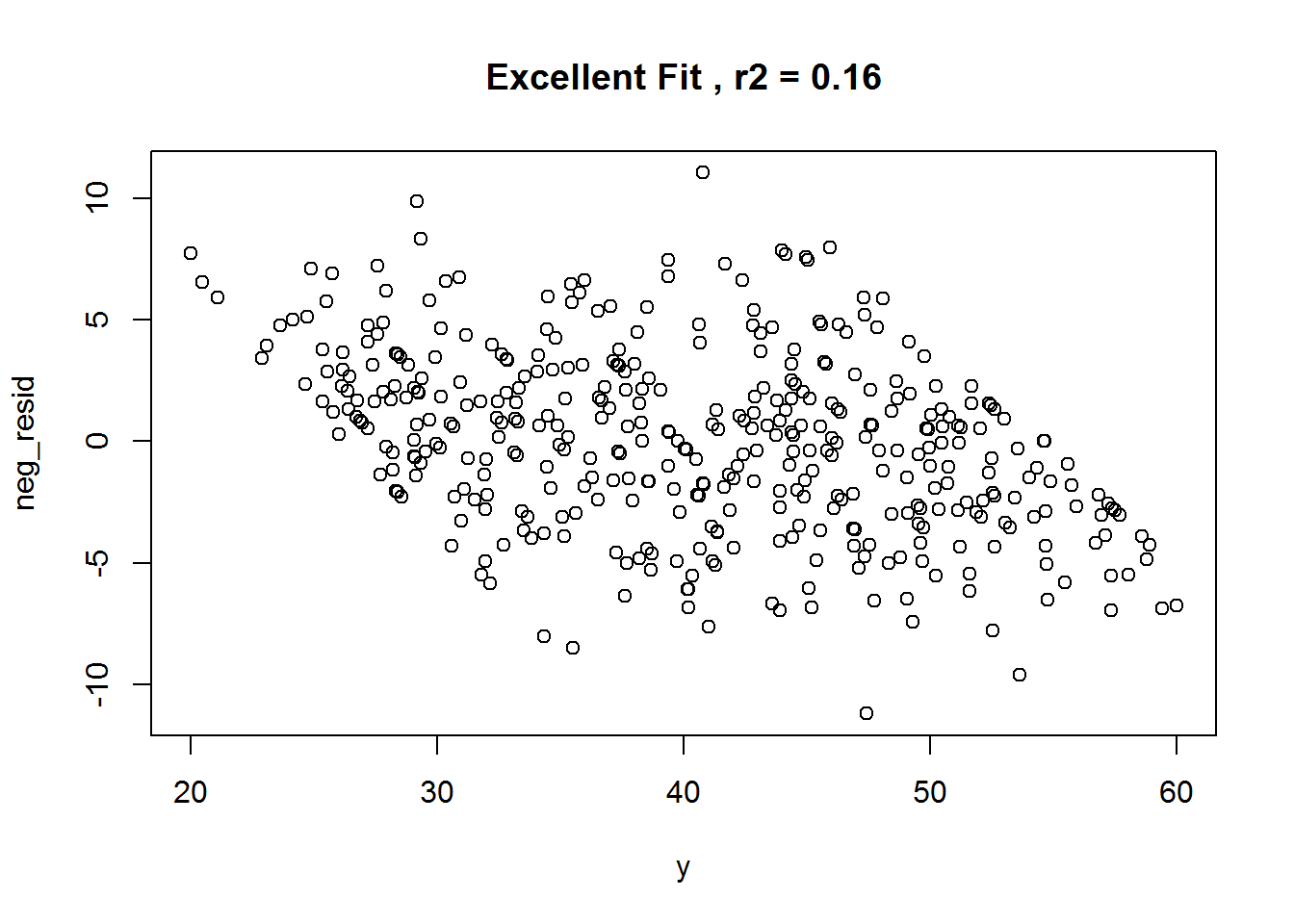


**Supplementary Figure S2.** Simplified simulation framework for BrainAGE.

**Supplementary Figure S3.** Linear and nonlinear age basis functions. $f_{1}\left( A \right)=100-A;f_{2}\left( A \right)= a\sqrt{b^{5}-A^{5}};f_{3}\left( A \right)=\frac{{100}^{2}c}{A^{2}}+d$. The constant $a, b, c$ and $d$ were chosen so that the range of all basis functions are approximately (20, 80): $a=\frac{1}{424},b=90, c=2.6, d=16$.

**Supplementary Figure S4.** Observed correlations between variables of interest and age in the T1000 dataset. The strongest correlations are close to 0.3 and include, for examples, PROMIS physical function (r=-0.33), waist circumference (r = 0.29), and sensation seeking (r=-.23).


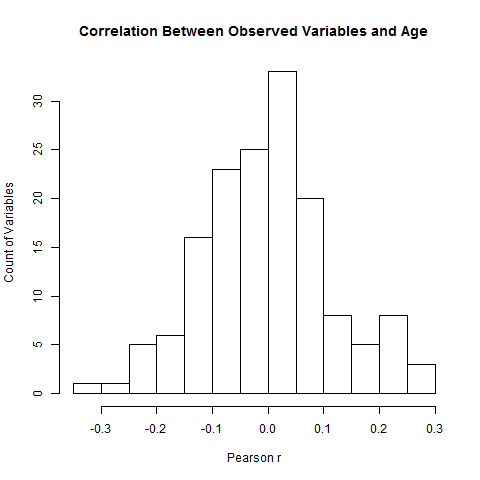


**Supplementary Table 1.** Coefficient structure in the derivation of 16 covariate types from the age-basis-functions in one simulation ($Z_{ij}\left( A \right)=\sum_{m=1}^{3} \alpha_{mj}f_{m}\left( A \right) + {\epsilon_{z}}_{i})$. While several particular $\alpha_{mj}$ coefficients are set to 0, others are randomly drawn from the uniform distribution $U(-2,-1)$. The covariates are then scaled so that the sum of coefficient values for one covariate equal -1.

| $\boldsymbol{j (}\boldsymbol{Z}_{\boldsymbol{j}}\boldsymbol{)}$ | $\boldsymbol{\alpha}_{\boldsymbol{1}\boldsymbol{j}}$ | $\boldsymbol{\alpha}_{\boldsymbol{2}\boldsymbol{j}}$ | $\boldsymbol{\alpha}_{\boldsymbol{3}\boldsymbol{j}}$ |
| --- | --- | --- | --- |
| 1 | 0 | 0 | 0 |
| 2 | -1 | 0 | 0 |
| 3 | 0 | -1 | 0 |
| 4 | 0 | 0 | -1 |
| 5 | 0 | -0.592 | -0.408 |
| 6 | -0.496 | 0 | -0.504 |
| 7 | -0.404 | -0.596 | 0 |
| 8 | -0.286 | -0.423 | -0.291 |
| 9 | 0 | 0 | 0 |
| 10 | -1 | 0 | 0 |
| 11 | 0 | -1 | 0 |
| 12 | 0 | 0 | -1 |
| 13 | 0 | -0.610 | -0.390 |
| 14 | -0.588 | 0 | -0.412 |
| 15 | -0.477 | -0.523 | 0 |
| 16 | -0.357 | -0.392 | -0.251 |

**Supplementary Table 2.** Proportion of p-values less than 0.05 for each covariate in 1,000 simulations of each type (small and large effect). For example, consider variable #2 that is related to the brain volumetric features: including age as a covariate increases the true positive rate for this variable from 0.329 to 0.468 in the small effect simulation. Similarly, for variable #10 that is NOT related to the brain volumetric features, including age as a covariate decreases the false positive rate for this variable from 0.920 to 0.042 in the large effect simulation.

| Covariate | Influences imaging features | Small effect  No Age | Small effect  With Age | Large effect  No Age | Large effect  With Age |
| --- | --- | --- | --- | --- | --- |
| 1 | TRUE | 0.330 | 0.419 | 0.523 | 0.695 |
| 2 | TRUE | 0.329 | 0.468 | 0.189 | 0.709 |
| 3 | TRUE | 0.129 | 0.389 | 0.253 | 0.658 |
| 4 | TRUE | 0.226 | 0.747 | 0.119 | 0.911 |
| 5 | TRUE | 0.085 | 0.611 | 0.066 | 0.822 |
| 6 | TRUE | 0.244 | 0.599 | 0.137 | 0.817 |
| 7 | TRUE | 0.109 | 0.400 | 0.059 | 0.691 |
| 8 | TRUE | 0.122 | 0.573 | 0.074 | 0.811 |
| 9 | FALSE | 0.060 | 0.049 | 0.061 | 0.047 |
| 10 | FALSE | 0.891 | 0.044 | 0.920 | 0.042 |
| 11 | FALSE | 0.140 | 0.059 | 0.147 | 0.059 |
| 12 | FALSE | 0.771 | 0.111 | 0.818 | 0.107 |
| 13 | FALSE | 0.449 | 0.070 | 0.499 | 0.069 |
| 14 | FALSE | 0.797 | 0.062 | 0.830 | 0.061 |
| 15 | FALSE | 0.572 | 0.040 | 0.607 | 0.043 |
| 16 | FALSE | 0.583 | 0.065 | 0.620 | 0.065 |
